# Incidental perioperative detection of congenital methemoglobinemia: A prospective case series from a South Indian referral centre

**DOI:** 10.46989/001c.164967

**Published:** 2026-07-31

**Authors:** Pooja Pushparaj, Aboobacker Mohamed Rafi

**Affiliations:** 1 Jubilee Mission Medical College and Research Institute, Thrissur, Kerala, India

**Keywords:** methemoglobinemia, Cytochrome B5 reductase deficiency, cyanosis, oximetry, incidental findings

## Abstract

**Background:**

Congenital methemoglobinemia is a rare inherited disorder caused by impaired reduction of methemoglobin to functional hemoglobin, resulting in functional hypoxia despite normal arterial oxygen tension. Due to its often-mild clinical phenotype, diagnosis is frequently delayed or made incidentally.

**Methods:**

This prospective case series of surgical patients was conducted at a tertiary care center in South India over a one-year period. Patients incidentally diagnosed with methemoglobinemia during perioperative or acute medical evaluation were included. Diagnosis was established using arterial blood gas analysis with co-oximetry and confirmed biochemically by erythrocyte NADH–cytochrome b5 reductase activity.

**Results:**

Ten surgical patients were identified, all originating from a single geographic region. Methemoglobin levels ranged from 14.4% to 29.1%. Most patients were asymptomatic or mildly symptomatic, with diagnosis prompted by refractory hypoxemia and a characteristic saturation gap. Enzyme assays confirmed Type I congenital methemoglobinemia in all cases. Management was largely supportive, with methylene blue reserved for symptomatic individuals.

**Conclusions:**

This case series highlights incidental perioperative detection as a key diagnostic opportunity and suggests possible regional clustering of congenital methemoglobinemia. Increased clinical awareness and targeted screening may improve the diagnosis of this underrecognized condition.

## INTRODUCTION

Methemoglobinemia is a rare blood disorder in which hemoglobin’s ferrous iron (Fe^2+^) is oxidized to the ferric state (Fe^3+^).^1–3^ Methemoglobin concentrations are typically maintained at below 1% under standard physiological conditions through endogenous reduction processes.^1^

The introduction of ferric iron causes allosteric modifications in the hemoglobin tetramer, resulting in a leftward shift of the oxygen-dissociation curve.^1^ This leftward shift significantly enhances the affinity of residual ferrous iron for oxygen, which subsequently hampers the effective release of oxygen to peripheral tissues.^1,4^ As a result, patients develop functional hypoxia and cellular ischemia even when arterial oxygen partial pressure (PaO_2_) is normal.^1^

Hereditary methemoglobinemia is most commonly attributable to a deficiency of NADH-cytochrome b5 reductase (CYB5R), which is encoded by the CYB5R3 gene and inherited in an autosomal recessive manner.^1,4^ This condition is divided into Type I and Type II, depending on tissue distribution.^1,4^

Type I disease affects only erythrocytes, causing persistent cyanosis, with patients otherwise asymptomatic or experiencing mild fatigue or headaches.^1,4^ In contrast, Type II disease impacts all cell types, resulting in pronounced neurological deficits, cognitive impairment, and a markedly decreased life expectancy.^1,4^ A key sign is central cyanosis that does not improve with oxygen, along with “chocolate brown” blood.^1,5^

Diagnosing methemoglobinemia is challenging because it often resembles cardiopulmonary disease.^1,6^ This condition is commonly identified incidentally during perioperative management, typically when pulse oximetry demonstrates persistent hypoxemia (usually around 85%) that does not respond to supplemental oxygen administration.^1,7^

The saturation gap is a key diagnostic indicator, referring to when peripheral oxygen saturation (SpO_2_) differs from calculated arterial oxygen saturation (SaO_2_) by more than 5%.^5,7^

The incremental contribution of this report lies in the prospective identification, biochemical confirmation in all cases, and documentation of a geographically aggregated series from a single district in India.

The primary objective of this study is to report the clinical spectrum of incidentally detected cases from a South Indian referral centre and to describe a hypothesis-generating observation of geographic aggregation identified during prospective perioperative case detection.

## METHODS

This prospective case series was conducted between November 2023 and November 2024 at a tertiary care teaching hospital in South India. Written informed consent for publication was obtained from all patients or their legal guardians.

Biochemical confirmation was performed by measurement of erythrocyte NADH–cytochrome b5 reductase activity at a national reference laboratory. Demographic data, clinical presentation, methemoglobin levels, management, and outcomes were recorded.

### Case ascertainment and study design

In our institution, all suspected cases of methemoglobinemia identified during perioperative evaluation are referred to the Department of Transfusion Medicine for diagnostic confirmation, including co-oximetry interpretation and biochemical testing. As part of this centralized consultation pathway, the authors were involved in the evaluation of all perioperative referrals for suspected methemoglobinemia during the study period.

Prospective identification was operationalized through real-time consultation requests from anesthesiology and surgical teams when patients demonstrated unexplained perioperative hypoxemia, defined as persistently low peripheral oxygen saturation (SpO₂) despite supplemental oxygen in the presence of normal or near-normal arterial oxygen tension (PaO₂).

Diagnosis was established based on arterial blood gas analysis with co-oximetry demonstrating elevated methemoglobin levels in the presence of normal arterial oxygen tension [3,8].

All referred patients were evaluated at presentation and underwent arterial blood gas analysis with co-oximetry as part of standard perioperative care. This series comprises consecutive surgical cases that meet the predefined inclusion criteria during the study period. No eligible patients with the defined biochemical findings were excluded.

Inclusion criteria were:

1. Surgical patients undergoing elective or emergency procedures;
2. Incidental perioperative detection of elevated methemoglobin levels on arterial blood gas co-oximetry;
3. Normal or near-normal PaO₂; and
4. Biochemical confirmation of reduced erythrocyte NADH–cytochrome b5 reductase activity.

Exclusion criteria included known cardiopulmonary disease accounting for hypoxemia, documented hemoglobinopathies, and clear recent exposure to oxidant drugs known to cause acquired methemoglobinemia.

Clinical, anesthetic, and biochemical data were recorded prospectively at the time of the perioperative consultation using a standardized data-collection format maintained by the transfusion medicine service.

Molecular genetic testing for CYB5R3 mutations was not performed due to limited access and cost constraints during the study period.

## RESULTS

All 10 cases in this series were detected in surgical patients during perioperative evaluation, including both elective and emergency procedures.

The age range was 9 months to 75 years, with a male predominance.

**Table 1.** Summary of Clinical and Biochemical Features

Sl No.	Age/Gender	Admission Reason	Key Symptoms/Cyanosis	Initial MetHb (%)	SpO₂ (%)	Saturation Gap (%)	NADH-CYB5R Activity (U/g Hb)	Treatment
1	20/M	Discectomy	Asymptomatic	16.8	91	7.6	2.51	Ascorbic acid, IVF
2	18/M	Appendicectomy	Dyspnoea, Fatigue, Cyanosis	19.7	80	19.2	4.34	Ascorbic acid, IVF
3	75/F	DKA/UTI – Diabetic Foot Surgery	Dyspnoea, Fatigue	22.0	88	11.3	5.01	Ascorbic acid, IVF, MB
4	49/M	Hernia Repair	Dyspnoea, Dyspnoea on exertion, Headache	20.6	91	6.7	6.87	Ascorbic acid
5	14/M	Appendicectomy	Dyspnoea, Cyanosis	29.1	72	13.0	3.25	Ascorbic acid, IVF, MB
6	16/F	Fibroadenoma Excision	Dyspnoea on exertion	27.4	88	6.0	5.92	Ascorbic acid
7	35/F	Vocal Cord Excision	Asymptomatic, Pallor	14.4	93	9.9	5.10	Ascorbic acid
8	43/F	AUB (Surgery)	Fatigue	19.5	96	7.0	4.55	Ascorbic acid, IVF
9	22/F	Appendicitis	Dyspnoea on exertion, Cyanosis	25.8	88	9.8	3.78	Ascorbic acid
10	9 months/ M	Cleft Lip Repair	Asymptomatic	20.0	90	7.0	4.11	O₂ Support

Values are presented as individual case data.

Methemoglobin levels were measured by arterial blood gas analysis with co-oximetry.

NADH–cytochrome b5 reductase activity was measured at a national reference laboratory.

SpO₂ refers to peripheral oxygen saturation measured by pulse oximetry.

PaO₂ refers to partial pressure of oxygen measured on arterial blood gas analysis.

The saturation gap represents the difference between pulse oximetry oxygen saturation and arterial oxygen saturation calculated by co-oximetry.

All patients were diagnosed with Type I (erythrocyte-restricted) congenital methemoglobinemia.

Reference range for erythrocyte NADH–cytochrome b5 reductase activity: approximately 30–40 IU/g Hb (laboratory-specific).

Abbreviations:

SpO₂, peripheral oxygen saturation; PaO₂, partial pressure of arterial oxygen; MetHb, methemoglobin; NADH-CYB5R, NADH–cytochrome b5 reductase; DKA, diabetic ketoacidosis; UTI, urinary tract infection; IVF, intravenous fluids.

### Perioperative and anesthetic context

Among surgically managed patients, both general and regional anesthesia were represented. In several cases, persistent low SpO₂ readings on pulse oximetry delayed induction or escalated oxygen therapy before the saturation gap was recognized. No procedures were canceled; however, delayed recognition resulted in additional investigations and prolonged perioperative monitoring in some cases. No perioperative adverse outcomes occurred.

All cases in this series were detected during perioperative evaluation, reinforcing the operating room as a key setting for recognition of congenital methemoglobinemia.

All patients originated from the same district. Most patients were asymptomatic or reported mild symptoms such as exertional dyspnea or fatigue. Visible cyanosis was present in four cases. SpO₂ ranged from 72% to 88%, with poor response to supplemental oxygen. Arterial blood gas analysis revealed normal PaO₂ values, resulting in a significant saturation gap.

Both elective and emergency procedures were represented. The majority of patients underwent general anesthesia, while a smaller number were evaluated preoperatively prior to induction.

In several cases, persistent low pulse oximetry readings despite supplemental oxygen initially raised concern for cardiopulmonary pathology or equipment-related artifact, prompting escalation of oxygen delivery and additional monitoring before methemoglobinemia was considered. Recognition of a saturation gap on arterial blood gas analysis clarified the diagnosis and prevented further unnecessary perioperative investigations.

No documented exposure to known oxidant drugs was identified in the perioperative period. Methemoglobin levels ranged from 14.4% to 29.1%. All patients demonstrated markedly reduced erythrocyte NADH–cytochrome b5 reductase activity, consistent with Type I congenital methemoglobinemia.

The median methemoglobin level was 20.3%, and the median SpO₂ at presentation was 89%.

### Management approach

Management decisions were guided by a symptom-based clinical framework, consistent with published recommendations for Type I congenital methemoglobinemia. Asymptomatic or mildly symptomatic patients were managed conservatively with patient education, avoidance of oxidant drugs, and oral ascorbic acid supplementation.

Methylene blue was reserved for 2 patients with significant hypoxia-related symptoms and/or elevated methemoglobin levels, for whom clinical concern warranted active intervention. Glucose-6-phosphate dehydrogenase (G6PD) status was not routinely assessed prior to methylene blue administration, as both treated patients were adults with no clinical or laboratory features suggestive of hemolysis, and received standard low-dose therapy without adverse events. No institutional protocol specific to congenital methemoglobinemia was in place during the study period.

Two symptomatic patients received methylene blue with prompt clinical improvement. No adverse outcomes were observed.

## DISCUSSION

This case series includes a clinically heterogeneous cohort with respect to age and surgical context, ranging from infancy to 75 years, and encompassing both elective and emergency surgical procedures. Although all patients were identified during perioperative evaluation, the breadth of age groups and operative urgency limits direct comparison across cases.

Given the small sample size, formal subgroup analysis was not feasible. However, descriptive subgrouping highlights that both pediatric and adult patients, as well as elective and emergency surgical cases, demonstrated similar diagnostic features, particularly persistent perioperative hypoxemia with a characteristic saturation gap. This heterogeneity reflects real-world perioperative practice, where congenital methemoglobinemia is often detected incidentally rather than through targeted screening.

A key finding of this prospective case series is the substantial incidence of incidental perioperative identification of congenital methemoglobinemia among patients with no prior awareness of their diagnosis.^1,8,9^

All cases showed markedly decreased NADH-cytochrome b5 reductase (CYB5R) activity, confirming a diagnosis of Type I recessive congenital methemoglobinemia.^1,4,6^

Clinically, the group showed a mild phenotype, with persistent central cyanosis frequently overlooked or misdiagnosed until perioperative stress revealed it.^1,4,6^

Although methemoglobin levels ranged from 14.4% to 29.1%, these individuals showed few symptoms, typical of the erythrocytic-limited form, in which life expectancy is usually unaffected.^1,6^ This stability shows how well these patients physiologically compensate, often staying active despite chronic functional anemia.^1,3,6^

Our results are consistent with research from India and international perioperative studies.^2,10^

In our study, the main diagnostic clue was always the saturation gap, as defined as a difference of over 5% between the SpO_2_ reading from pulse oximetry and the SaO_2_ value obtained from arterial blood gas analysis.^7,9,11^

This phenomenon, together with the distinctive “chocolate brown” coloration of arterial blood, has been thoroughly documented in various case series as a principal indicator for diagnosing the disorder in acute care environments.^3,7,11^

Notably, pulse oximetry readings often remained near 85% even with high oxygen levels, consistent with classic methemoglobin interference at 660 nm and 940 nm wavelengths.^1,6,11^

These findings have important clinical relevance, especially for anesthesiologists and critical care doctors, who are frequently the initial healthcare professionals to treat these patients.^3,8,9^

The perioperative period is a crucial time for diagnosing, as general anesthesia and the need for precise oxygen monitoring highlight the saturation gap.^9^

Identifying this gap helps prevent needless tests, like echocardiography or pulmonary imaging, that often occur when cardiopulmonary causes of cyanosis are wrongly assumed.^6^

These observations highlight how perioperative pulse oximetry limitations can lead to diagnostic uncertainty and procedural delays, underscoring the importance of early recognition of the saturation gap in anesthetic practice.

Awareness helps avoid unnecessary increases in oxygen therapy or mechanical ventilation when low SpO_2_ is due to methemoglobin’s absorbance limits.^1,6^

Molecular studies from India, including the work by Warang et al., demonstrate recurrent CYB5R3 mutations associated with Type I congenital methemoglobinemia, providing biological plausibility; however, these findings cannot be extrapolated to our cohort in the absence of molecular testing.^10^

All cases in this series originated from a single geographic district in South India. While this aggregation is noteworthy, it cannot be interpreted as evidence of true regional clustering or genetic predisposition. The small sample size, absence of population denominator data, lack of comparison with neighboring districts, and absence of molecular genetic confirmation substantially limit epidemiologic inference.

Several alternative explanations are plausible, including referral bias to a tertiary center, heightened local clinical awareness, and availability of co-oximetry and biochemical enzyme testing, which may preferentially identify cases within this catchment area. Mild phenotypes of Type I congenital methemoglobinemia may remain undiagnosed in other regions where diagnostic suspicion or laboratory access is limited.

Therefore, the observed geographic aggregation should be regarded as hypothesis-generating rather than indicative of a true epidemiologic concentration or founder effect. Larger, population-based studies incorporating molecular characterization are required to determine whether true regional or genetic clustering exists.

Molecular confirmation was not performed for these patients, so the idea of a regional genetic cluster remains hypothetical and requires further genomic study.^6^

The observed clustering may result from under-recognition in other regions, where patients with mild cyanosis might not receive the necessary biochemical tests for diagnosis.^4,12^

Management followed symptom-based rather than protocol-driven thresholds, consistent with published recommendations for Type I congenital methemoglobinemia.

Type I congenital methemoglobinemia is usually managed conservatively, since it is mainly a cosmetic issue, unless acute stressors are present.^1,4^

Ascorbic acid (200–1000 mg/day) effectively and safely reduces cyanosis by non-enzymatic methemoglobin reduction.^1,7,11^

Methylene blue (1–2 mg/kg) is preferred for treating acute symptoms or when methemoglobin levels are above 30%. However, it should be administered very carefully in infants or patients who may have G6PD deficiency, as it can cause hemolysis.^1,7,11^

While G6PD testing is recommended prior to methylene blue administration when feasible, particularly in high-risk populations, no hemolytic complications were observed in this series. This highlights the need for standardized perioperative protocols and improved access to rapid G6PD testing in resource-limited settings.

Patient education is crucial; individuals should avoid known oxidant triggers, including benzocaine, lidocaine, and antibiotics like dapsone.^1,3,4,7,11^ Providing patients with a medical alert system can prevent life-threatening acquired exacerbations during future medical encounters.^1,4^

This case series shows that, despite stable hemoglobin-oxygen readings, underlying molecular issues may exist, requiring clinicians to stay alert for discrepancies between measured and actual function.^1,11^

## LIMITATIONS

This study is limited by its small sample size and single-center design, which restricts generalizability and precludes epidemiologic inference.

It also lacks molecular genetic confirmation. Nevertheless, biochemical confirmation in all cases and consistent clinical features strengthen the observations.

The small sample size and lack of population-level denominator data prevent assessment of disease prevalence or true geographic clustering; the observed aggregation may reflect institutional referral patterns rather than underlying epidemiology.

Although all cases were biochemically confirmed by reduced erythrocyte NADH–cytochrome b5 reductase activity, the absence of CYB5R3 molecular testing limits conclusions regarding definitive congenital etiology, founder effects, and comparison with known Indian mutation spectra. Biochemical confirmation alone cannot fully exclude rare chronic acquired causes of methemoglobinemia. A detailed family history focusing on symptoms suggestive of methemoglobinemia was obtained; however, no affected relatives were identified. Formal pedigree construction and family screening were not performed, and molecular testing was not feasible due to limited access, cost, and logistical constraints.

Future studies incorporating molecular characterization and population-level data are required to clarify genetic and regional patterns.

## CONCLUSION

Congenital methemoglobinemia remains an underrecognized cause of unexplained hypoxemia.

This case series documents a hypothesis-generating geographic aggregation observed during prospective perioperative detection and underscores the need for population-based and molecular studies.

This series adds prospective, biochemically confirmed data from an underreported region and underscores perioperative monitoring as a critical diagnostic opportunity.

### Authors’ Contributions - CRediT

Conceptualization: Pooja Pushparaj, Aboobacker Mohamed Rafi;

Methodology: Pooja Pushparaj, Aboobacker Mohamed Rafi;

Investigation: Pooja Pushparaj;

Data curation: Pooja Pushparaj;

Formal analysis: Pooja Pushparaj, Aboobacker Mohamed Rafi;

Validation: Aboobacker Mohamed Rafi;

Visualization: Pooja Pushparaj;

Writing – original draft preparation: Pooja Pushparaj;

Writing – review & editing: Aboobacker Mohamed Rafi;

Supervision: Aboobacker Mohamed Rafi;

Project administration: Aboobacker Mohamed Rafi;

Resources: Aboobacker Mohamed Rafi.

### Competing Interests

The authors declare that they have no competing interests.

### Ethical Conduct Approval

The study was conducted in accordance with the ethical principles of the Declaration of Helsinki. Written informed consent was obtained from all participants/legal guardians prior to inclusion in the study.

### Patient Consent Statement

Appropriate written informed consent for publication was obtained from the patient(s)/guardian(s).

## Supplementary Material

Supplementary Case Distribution Geographic distribution of patients with congenital methemoglobinemia

## Data Availability

The data are available from the corresponding author upon reasonable request.
