# Supplementary material for: Incidental perioperative detection of congenital methemoglobinemia: A prospective case series from a South Indian referral centre
Source: Clin Hematol Int. 2026 Jul 31;8(3):10–7. doi: 10.46989/001c.164967 (PMC13428680; doi:10.46989/001c.164967)
Supplement: Supplementary Case Distribution — Geographic distribution of patients with congenital methemoglobinemia [file chi_2026_8_3_164967_353361.pdf]

Supplementary Case Distribution

Figure 1

Geographic distribution of patients with congenital methemoglobinemia

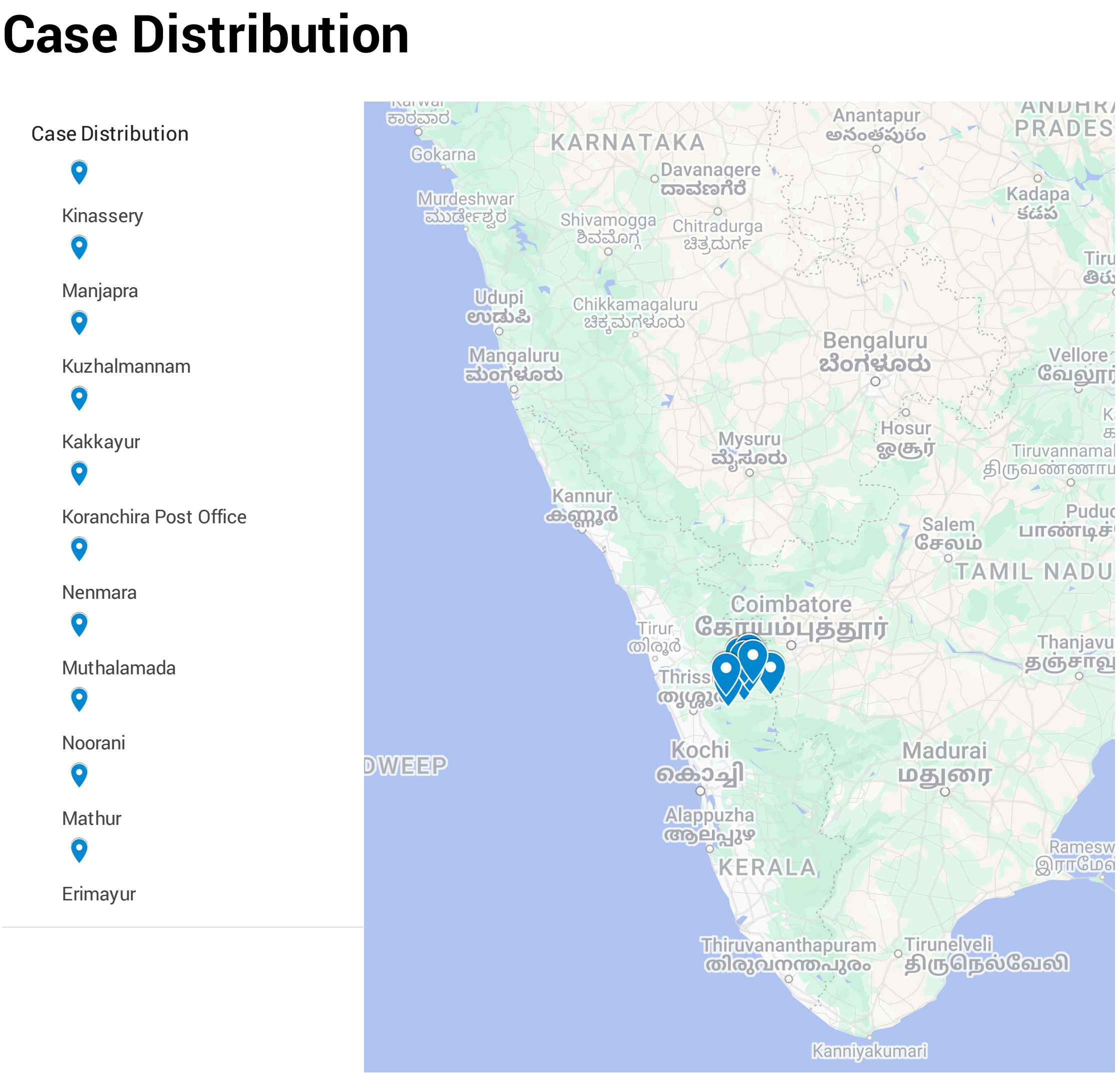

Legend:

Map illustrating the residential locations of patients included in the case series, demonstrating clustering within a single district in Kerala, South India. Individual locations are shown without identifiable patient information.

This figure is intended for descriptive visualization only and does not imply epidemiologic clustering.
